# Ruling in, ruling out: the clinical utility of plasma biomarkers in diagnosis of Alzheimer’s disease

**DOI:** 10.1172/JCI198725

**Published:** 2025-10-01

**Authors:** Julie K. Wisch, Beau M. Ances

**Affiliations:** Department of Neurology, Washington University in St. Louis, St. Louis, Missouri, USA.

## Introduction

On May 16, 2025, the US FDA approved the first blood-based test for amyloid plaques — the Lumipulse G pTau217/β-Amyloid 1–42 Plasma Ratio — for adults aged 55 years and older who are exhibiting signs and symptoms of Alzheimer disease (AD) (1).

The presence of amyloid plaques has long been recognized as the earliest pathological indicator of looming cognitive decline and functional impairment associated with AD. First documented by Alois Alzheimer in 1906 (2), the field of AD research was hampered by limitations in their detectability: amyloid plaques could only be identified at autopsy. Nearly 100 years later, a radiotracer known as the Pittsburgh Compound B (PiB) was the first approved in vivo approach for visualizing and quantifying amyloid plaques via PET imaging (3). The challenges, costs, and lack of availability of PET led to development of cerebrospinal fluid (CSF) assays, with the first approval occurring in 2022 (4). The development of a blood-based test for AD pathology has long been an aim of the field; the possibility was first suggested in 1984 by Glenner and Wong, who isolated the amyloid protein (5). The successful development of a blood-based assay has the potential to transform screening for AD pathology, expanding accessibility and allowing for application in nonspecialized clinical practice.

Diagnosing AD can be difficult owing to variable clinical presentation and the presence of copathologies. In specialized memory centers, clinical diagnosis rates based on patient history and cognitive testing alone range from 70% to 87% sensitivity and 44% to 71% specificity (6), suggesting diagnostic accuracies of 50%–75% compared with assessment at autopsy. Adding amyloid PET imaging to clinical evaluations increases diagnostic sensitivity to 79%–98% and specificity to 76%–100% (7), increasing the estimated diagnostic accuracy to between 78% and 98%. Methods that increase access to amyloid status substantially enhance diagnostic precision and ensure that available antiamyloid therapies are given to appropriate individuals. A blood-based alternative to PET or CSF could further expand access to biomarker data, reduce misdiagnosis, and improve patient care.

## Biological context

β-Amyloid 1–42 is a core component of amyloid plaques (5), and its accumulation in the brain is an early hallmark of AD pathology. As amyloid accumulates, it triggers downstream processes, including the hyperphosphorylation of tau proteins at more than 40 known amino acid sites (8) — events that have been quantified in both CSF (9, 10) and, more recently, in plasma (11). Longitudinal work has demonstrated that the association between PET change rates for amyloid and tau is largely mediated by soluble tau, indicating that tau phosphorylation represents a critical step in the transition from amyloid accumulation to the development of tau tangles (8). Among the many phosphorylated tau species, plasma pTau217 — measuring phosphorylation at amino acid site 217 — has emerged as a leading biomarker owing to its strong and early association with amyloid pathology (12). Despite its name, pTau217 does not reflect tau tangle burden. Rather, it rises in response to amyloid plaque formation and appears to be more responsive to the presence of amyloid plaques than neurofibrillary tangles, making it a reliable marker of amyloid-related disease activity (13, 14).

## Underlying studies

In the clinical study submitted with the application (1), 499 individuals with either cross-sectional paired plasma and amyloid PET measurement or cross-sectional plasma and CSF quantification were assessed using a two-threshold model that classified individuals as low, high, or indeterminate for amyloid positivity. This approach, widely applied in the field, is designed to maximize both sensitivity and sensitivity while identifying individuals who would benefit from additional screening (i.e., PET or CSF) (15). In this cohort, the positive predictive value was 91.7% and the negative predictive value was 97.3%, while roughly 20% of individuals landed within the indeterminate zone, suggesting that they would need to be referred for further clinical testing. However, the full FDA review is not publicly available, limiting independent evaluation beyond the press release.

Outside of the FDA submission, this assay has been evaluated in multiple research settings. The largest published study (*n =* 1767) compared plasma pTau217 and the pTau217/β-amyloid 1–42 (pTau217/Aβ42) ratio to CSF Aβ42/pTau181 in 5 European cohorts, yielding positive predictive values of 89%–95% and negative predictive values of 77%–90%. When using plasma pTau217 alone 12%–17% of individuals were classified as indeterminate, compared with 7%–10% when the plasma pTau217/Aβ42 ratio was employed (16). Similar performance in other studies using CSF as the standard (AUC = 0.90–0.97, refs. 17–19) as well as in comparisons to amyloid PET imaging (AUC = 0.94–0.96, ref. 20) have been observed. Studies using the two-threshold approach report indeterminate rates of 12%–18% (21, 22). Although evaluation of the performance of plasma pTau217 alone rather than the ratio of pTau217/Aβ42 is the predominant means by which the Lumipulse assay has been evaluated in the literature, the findings broadly suggest that the performance of pTau217/Aβ42 is similar to pTau217 alone while greatly decreasing the number of individuals who receive an indeterminate designation.

While multiple assays have been developed, the Lumipulse G pTau217/β-Amyloid 1–42 Plasma Ratio is the first to receive approval. In one head-to-head comparison of some of the most common pTau217 assays, the Lumipulse pTau217 plasma assay (AUC = 0.896, 95% CI: 0.864, 0.928) outperformed assays developed by ALZpath (AUC = 0.885, 95% CI: 0.851, 0.920) and Janssen (AUC = 0.882, 95% CI: 0.848, 0.916), but not C2N (AUC = 0.927, 95% CI: 0.900, 0.955) (12). The Alzheimer’s Association Global Biomarker Standardization Consortium conducted the broadest evaluation of the developed plasma assays, evaluating the fold change in CSF-confirmed AD cases versus individuals acting as controls, considering 33 different assays. The Lumipulse pTau217 plasma assay generated one of the highest median fold changes (5.69), indicating that the presence of AD pathology triggers a substantive change in circulating levels of plasma pTau217 (23). However, the pTau217/Aβ42 ratio was not specifically included in these analyses.

Importantly, while current evidence supports the diagnostic utility of plasma pTau217, the ratio of plasma pTau217/Aβ42 has received considerably less attention in the literature. Further, longitudinal data remains limited. Such evidence is essential for clinical interpretation, especially in the context of monitoring disease progression and treatment response over time.

In one of the few published longitudinal studies, evidence suggests that plasma pTau217 is a dynamic biomarker that is responsive to the development of AD pathology over time, although again, it did not investigate the ratio of plasma pTau217/Aβ42. In this evaluation of 209 individuals with a mean follow-up time between samples of 2.1 years, amyloid-positive individuals displayed a higher annualized rate of change of plasma pTau217 than amyloid-negative individuals. Within the amyloid-negative cohort, apolipoprotein e4 carriers had higher mean plasma pTau217 (21).

A separate study using the Quanterix pTau217 assay examined short-term biological variation by measuring weekly plasma samples over 10 weeks in 20 healthy adults (age 40–60 years). Within-subject biological variation was 10.3% (95% CI: 9.2%–11.7%) and between-subject biological variation was 21.1% (95% CI: 15.1%–29.3%). Short term fluctuations of plasma pTau217 were apparent in some, but not all, individuals. Their analysis concludes that 3 samples would allow for estimation of the individual’s true homeostatic point within 5% of the true value (as compared with the roughly 20% error yielded with a single sample) (24). This finding has practical implications for clinical implementation, highlighting the potential value of repeat sampling to improve measurement precision in individual patients. This within-subject biological variation is likely to be assay dependent, and the results may not generalize to the recently approved Lumipulse G pTau217/β-Amyloid 1–42 Plasma Ratio; however, the methodological rigor and practical relevance of this study suggests that high-frequency sampling of relatively few individuals may provide key performance insights, and studies similar to this one may be highly informative for this newly approved assay.

## Implications for clinical practice

The FDA approval of the Lumipulse G pTau217/β-Amyloid 1–42 Plasma Ratio as a blood-based test for AD is likely to be just the first of many approvals, as the field of blood-based biomarkers in AD is rapidly expanding. More than 30 assays have been published in the literature (23), and it will be incumbent on physicians to keep track of the myriad advantages and disadvantages associated with each. For the use case outlined in the FDA approval, that this specific test is relevant for adults over the age of 55 exhibiting signs and symptoms of AD, the weight of evidence suggests that the test has a high degree of utility most particularly in ruling out AD as the source of observed clinical symptoms (97% negative predictive value) but also in suggesting that AD pathology is present (90% positive predictive value). This test will be informative for 4 in 5 patients, with the remaining 20% falling in an indeterminate zone. The bulk of the literature evaluating plasma pTau217 suggests referring these individuals to follow-up assessments like lumbar puncture or PET scanning; although work by Brum et al. is suggestive that multiple plasma evaluations could also suffice (24). A similar evaluation of the biological variation of the Lumipulse G pTau217/β-Amyloid 1–42 Plasma Ratio has not been released, but the field would benefit from this analysis, and a conclusion regarding the number of samples required to reliably estimate an individual’s true homeostatic level of circulating plasma pTau217 and β-amyloid 1–42.

Importantly, the Lumipulse G pTau217/β-Amyloid 1–42 Plasma Ratio is also only approved for initial assessment of the presence of amyloid plaques in the brain. With the recent approval of drugs such as donanemab (25) and lecanemab (26), antiamyloid therapies are being deployed in memory clinics across the country. Plasma biomarkers have not yet been approved for monitoring response to drug when there is a pharmacological intervention applied. Importantly, plasma pTau217 (but not the ratio of plasma pTau217/AB 42) has been collected in several clinical trials as researchers attempt to understand their utility in this context. In the TRAILBLAZER-ALZ trial, plasma pTau217 (Quanterix) showed a measurable response to donanemab as early as 12 weeks after initial dosing. Over the 76-week study, mean plasma pTau217 levels decreased by 23% from baseline in the treatment group and increased by 6% in the placebo group. Percentage change in amyloid PET signal was significantly correlated with percentage change in plasma pTau217 levels (R = 0.484, 95% CI: 0.359–0.592), suggesting that reductions in plasma pTau217 may reflect reductions in amyloid plaque burden (26). This suggests plasma pTau217 may be useful in monitoring response to drug; however, assay-specific validation is required.

## Conclusion

The recent FDA approval of the first blood-based test for amyloid pathology marks a major milestone in AD diagnostics. Incorporating blood-based biomarkers into clinical practice has the potential to enhance diagnostic accuracy while reducing patient burden. However, substantial work remains. Given the growing number of assays reported in the literature, additional regulatory approvals are likely, each with unique performance characteristics. Further research is needed to characterize intraindividual variability and assay-specific responses to antiamyloid therapies. Notably, limited attention has been given to individuals who fall within the indeterminate classification range (exceptions are found in refs. 27, 28) — an issue that will gain importance as therapeutic strategies shift toward earlier intervention. Ultimately, blood-based screening could play a critical role in ruling out AD or prompting clinical action, helping patients and their caregivers navigate the challenges of an AD diagnosis.

## Version 1 10/01/2025

Electronic publication
